# Hyperoside Nanomicelles Alleviate Atherosclerosis by Modulating the Lipid Profile and Intestinal Flora Structure in High-Fat-Diet-Fed Apolipoprotein-E-Deficient Mice

**DOI:** 10.3390/molecules28135088

**Published:** 2023-06-29

**Authors:** Yuwen Shi, Mengcheng Jiang, Yuhang Zhang, Yuanyuan Diao, Na Li, Weipeng Liu, Zhidong Qiu, Ye Qiu, Ailing Jia

**Affiliations:** Pharmacy College, Changchun University of Chinese Medicine, Changchun 130117, China; syuwen329@163.com (Y.S.); jmcjmc913@163.com (M.J.); zhangyuhangxz@163.com (Y.Z.); 15834653129@163.com (Y.D.); 15981526110@163.com (N.L.); 18715586624@163.com (W.L.); qzdcczy@163.com (Z.Q.)

**Keywords:** apolipoprotein E, atherosclerosis, hyperoside nanomicelle, intestinal flora, serum lipidomics

## Abstract

Atherosclerosis (AS) is a serious threat to human health and the main pathological basis of cardiovascular disease. Hyperoside (Hyp), a flavonoid found mainly in traditional Chinese herbs, can exert antitumor, anti-inflammatory, antioxidant, and cardiovascular-protective effects. Herein, we prepared hybrid nanomicelles (HFT) comprising Hyp loaded into pluronic F-127 and polyethylene glycol 1000 vitamin E succinate and assessed their effects on AS. To establish an AS model, apolipoprotein-E-deficient (ApoE^−/−^) mice were fed a high-fat diet. We then analyzed the effects of HFT on AS-induced changes in aortic tissues and metabolic markers, simultaneously assessing changes in gut flora community structure. In mice with AS, HFT significantly reduced the aortic plaque area; decreased levels of total cholesterol, triglyceride, low-density lipoprotein cholesterol, inflammatory factors, and inducible nitric oxide synthase (NOS); increased high-density lipoprotein cholesterol, endothelial NOS, superoxide dismutase, catalase, and glutathione levels; and promoted the proliferation of beneficial gut bacteria. HFT could regulate intestinal flora structure and lipid metabolism and inhibit inflammatory responses. These beneficial effects may be mediated by inhibiting nuclear factor kappa B signal activation, reducing inflammatory factor expression and improving gut microflora structure and dyslipidemia. The present study provides an empirical basis for the development and clinical application of new dosage forms of Hyp.

## 1. Introduction

Atherosclerosis (AS) is characterized by the accumulation of lipids or fibrous elements in the innermost layers of large- and medium-sized arteries [1]. AS is the main pathological basis of cardiovascular disease and the leading cause of morbidity and mortality worldwide. Approximately 20 million individuals die from atherosclerotic cardiovascular disease annually, accounting for 52% of all deaths [2]. Macrophage-related cholesterol metabolism and inflammation are the two major drivers of AS. In the presence of lipids, oxidized low-density lipoproteins (ox-LDL) are trapped in the intima, activating endothelial cells and leukocytes that can severely damage vascular tissue and promote the secretion of various harmful factors by endothelial cells, thereby inducing the formation of foam cells and fatty streaks [3,4]. During AS development, inflammation is mainly manifested via the activation of nuclear factor kappa B (NF-κB) and an increase in the expression of pro-inflammatory compounds [5]. The NF-κB pathway mediates inflammatory and metabolic responses [6]; hence, inhibiting the activation of the NF-κB pathway can reduce the inflammatory response, regulate lipid metabolism levels, and reduce lipid deposition. The NF-κB family has been shown to activate several genes associated with inflammation, which have crucial implications for understanding cardiovascular disease, colitis, autism spectrum disorders, and other neurodegenerative disorders [7,8,9]. Regarding the treatment of AS, statins (lipid-lowering drugs) can effectively reduce the risk of cardiovascular disease [10]. However, statins exert limited effects on pathogenic factors other than lipid metabolism and may cause myopathy, gastrointestinal diseases, kidney damage, liver damage, and fatigue [11,12,13]. Accordingly, identifying safe and effective alternative medications to treat patients with AS remains a critical challenge.

Hyperoside (Hyp; C_21_H_20_O_12_, molar mass = 464.38 g/mol) is mainly found in traditional Chinese herbs [14,15]. Hyp can exert anti-inflammatory, antioxidative, anticancer, anti-hyperglycemic, anticoagulant, and cardiovascular-protective effects, which are beneficial for regulating inflammatory responses [16,17]. Hyp has been shown to inhibit ox-LDL in vascular smooth muscle cells via the oxLDL-LOX-1-ERK pathway, thereby indicating the role of Hyp in treating and preventing atherosclerosis [18]. However, Hyp has poor water solubility and limited bioavailability [19]; therefore, further research is needed to establish an optimal dosage form for clinical application. In recent years, various anti-AS nanodrug delivery systems have been developed to improve the precision targeting and site-specific release capabilities of hydrophobic drugs [20]. Nanodrug delivery systems have broad application prospects in the treatment of AS [21]. Pluronic™ F-127 (F127) is an ABA-type triblock copolymer with excellent biocompatibility, high protein stability, and no myotoxicity or immunotoxicity [22]. D-α-Tocopherol polyethylene glycol 1000 succinate (TPGS) is a US Food and Drug Administration (FDA)-approved amphiphilic vitamin E succinate derivative, widely used in drug delivery systems as a drug adjuvant [23]. Previously, we, for the first time, encapsulated Hyp in pluronic F-127 (F127) and polyethylene glycol 1000 vitamin E succinate (TPGS) to prepare mixed nanomicelles (Hyp-F127/TPGS, HFT).

Accumulated evidence suggests that gut dysbiosis can promote the development of AS by regulating inflammatory processes and microbial metabolites, and that gut microbial metabolites from choline and phosphatidylcholine (PC) can directly increase the risk of AS [24,25]. Furthermore, consuming a high-fat diet (HFD)can interfere with gut microbiota composition, increase gut microbiota endotoxin production, enhance the expression of tumor necrosis factor (TNF)-α and inducible nitric oxide synthase (iNOS), and promote NF-κB activation [26]. Pro-inflammatory flora has been shown to facilitate systemic inflammation and accelerate AS in Ldlr^−/−^ mice [27]. Moreover, gut microbiota has been shown to impact the pathogenesis of AS by modulating circulating cholesterol levels [28]. Therefore, a notable connection exists between gut microbiota, inflammation, cholesterol metabolism, and AS. Gut microbiota composition is reportedly modulated by flavonoid–microbiota interactions, and flavonoids can play a therapeutic role in cytokine-induced intestinal inflammation [29]. Hao et al. have shown that Hyp was absorbed in both of the gastrointestinal regions of rats, although intestinal absorption was superior [30]. However, its effect on the intestinal flora has yet to be verified. Gut microbiota can also play a protective role in the development of AS by affecting lipid metabolism [31,32]. In addition to genomics and proteomics, lipidomics has emerged as a new branch of metabolomics, with comprehensive lipid profiling providing an opportunity to discover new biomarkers for specific diseases. It has been suggested that dyslipidemia and, consequently, AS could develop owing to an imbalance in lipid metabolites [33]. Meanwhile, elevated serum triglyceride (TG) and low-density lipoprotein cholesterol (LDL-C) levels have been identified as risk factors for AS [34].

As the active ingredient in most plants, Hyp has been extensively investigated to establish its pharmacological activities and mechanism of action. However, no previous study has reported the preparation of Hyp-mixed nanomicelles or the combined analysis of intestinal flora and serum lipidomics to elucidate the mechanism of Hyp-mediated anti-AS effects. Accordingly, using the combined analyses of gut microbiota and serum lipidomics, we aimed to determine the mechanism of HFT in alleviating AS. Moreover, the present study could help guide the identification of safe and effective alternative drugs for treating AS.

## 2. Results

### 2.1. Morphological Characterization of HFT

The resulting HFT solution was yellow and clear. Under laser irradiation, the solution demonstrated an obvious light path, exhibiting the Tyndall effect. According to a transmission electron microscopy (TEM; JEM-1200EX, JEOL, Tokyo, Japan), the HFT micelles were spherical, non-adhesive, morphologically intact, and uniformly distributed. Based on preliminary stability results, HFT was maintained for four weeks, appearing as a yellow, clear, transparent solution with no flocculent precipitation. Compared with day zero, entrapment efficiency and drug loading were minimally altered at four weeks. These findings indicated that the HFT was stable at a low temperature [35].

### 2.2. Effects of HFT on Body Mass and Aortic Plaques in ApoE^−/−^ Mice

**Figure 1:**
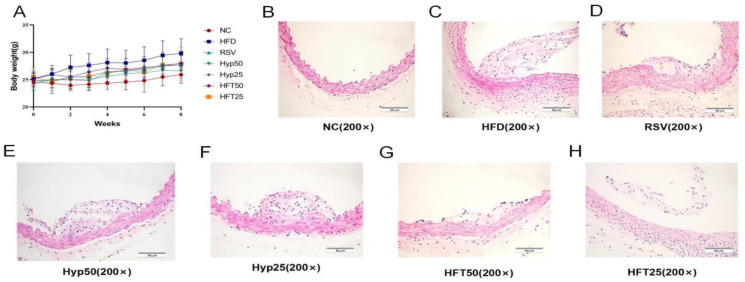
Effects of different doses of HFT treatment (eight weeks) on (**A**) the body weight of ApoE^−/−^ mice (*n* = 8 per group). (**B**–**H**) Hematoxylin–eosin staining of atherosclerotic plaque areas at 200× magnification.

The HFD group exhibited significantly higher body mass than the normal control (NC) group (*p* < 0.001). After 8 weeks, body mass was significantly reduced in the Hyp and HFT groups (*p* < 0.05), with the most notable reduction observed in the HFT50 group (Figure 1A), suggesting that treatment with Hyp and HFT could ameliorate AS by modulating body mass.

To verify the alleviating effect of Hyp and HFT on AS, we analyzed the aortic plaque areas of the groups. As shown in the hematoxylin–eosin (H and E)-stained sections presented in Figure 1B–H, the NC group displayed a smooth endothelium with no plaque formation. Regarding the HFD group, the endothelium was damaged, with evident plaque formation in the lumen. In the HFD + resveratrol (RSV) group, the luminal plaque area was reduced, the endothelium was rough, and the extent of intima-media damage was comparable with that observed in the HFD group. The luminal plaque area of the HFD + high-dose Hyp (Hyp50) group was reduced when compared with that of the HFD group, accompanied by a small number of foam cells. Compared with the HFD group, the low-dose HFT (HFT25) group exhibited a reduced plaque area, a less damaged endothelium, and a slightly thickened mesothelium.

### 2.3. Effects of HFT on Blood Lipid Levels in ApoE^−/−^ Mice

**Figure 2:**
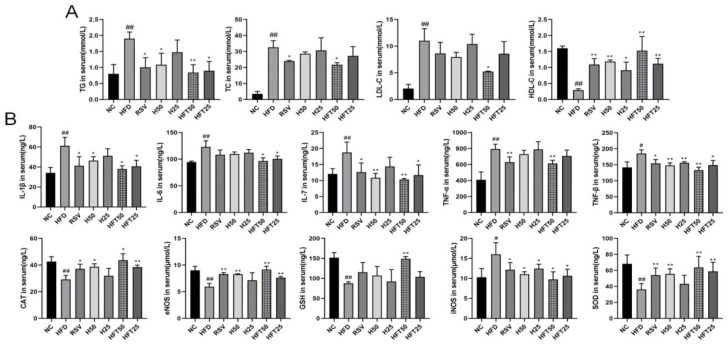
Effects of HFT on blood lipid levels in ApoE^−/−^ mice. Variations in (**A**) TG, TC, LDL-C, and HDL-C content; (**B**) IL-1β, IL-6, IL-7, TNF-α, TNF-β, CAT, eNOS, GSH, iNOS, and SOD levels (*n* = 3 per group). ^##^ *p* < 0.01 and ^#^ *p* < 0.05 compared with the NC group; ** *p* < 0.01 and * *p* < 0.05 compared with the HFD group.

Next, we analyzed AS-related lipid indices. Compared with the NC group, the HFD group exhibited elevated levels of TG, total cholesterol (TC), and LDL-C (*p* < 0.01) and a reduced level of high-density lipoprotein cholesterol (HDL-C) (*p* < 0.01). Compared with the HFD group, the RSV and Hyp50 groups had reduced TG (*p* < 0.05) and elevated HDL-C (*p* < 0.01) levels; in the HFD + high-dose HFT (HFT50) group, the levels of the four above-mentioned indicators varied from those in the HFD group (*p* < 0.05, *p* < 0.01). At the same dose, TC and LDL-C levels were lower in the HFT group than those in the Hyp group (*p* < 0.01; Figure 2A). These results suggested that HFT could reverse lipid abnormalities in AS.

Inflammation is closely related to AS; therefore, we analyzed the effect of the HFT intervention on inflammation. The HFD group had higher levels of pro-inflammatory factors than the NC group (*p* < 0.05 and *p* < 0.01, respectively). The HFT50 group exhibited the greatest decrease in levels of pro-inflammatory factors when compared with the HFD group and all treatment groups (*p* < 0.05, *p* < 0.01). The HFT50 group had lower levels of interleukin (IL)-1β, IL-6, and TNF-α than the Hyp group (*p* > 0.05), suggesting that a chronic inflammatory response accompanies the development of AS, and pharmacological interventions could reduce the expression of inflammatory factors. The HFT50 group had higher levels of endothelial nitric oxide synthase (eNOS), superoxide dismutase (SOD), catalase (CAT), and glutathione (GSH), in addition to iNOS, than the HFD group (Figure 2B). Reduced eNOS levels have been suggested as the main cause of endothelial dysfunction [36], which is consistent with the findings of the present study, thereby suggesting that AS may be related to oxidative stress damage. Therefore, we hypothesized that HFT could ameliorate AS by reducing inflammation, oxidative stress, and endothelial cell damage.

### 2.4. Effect of HFT on the Alpha and Beta Diversity of Intestinal Flora in ApoE^−/−^ Mice

Regarding intestinal flora, the HFD group had lower species abundance and diversity than the NC group. Drug intervention increased species abundance and diversity, although not significantly when compared with that of the HFD group (*p* > 0.05; Table 1).

**Figure 3:**
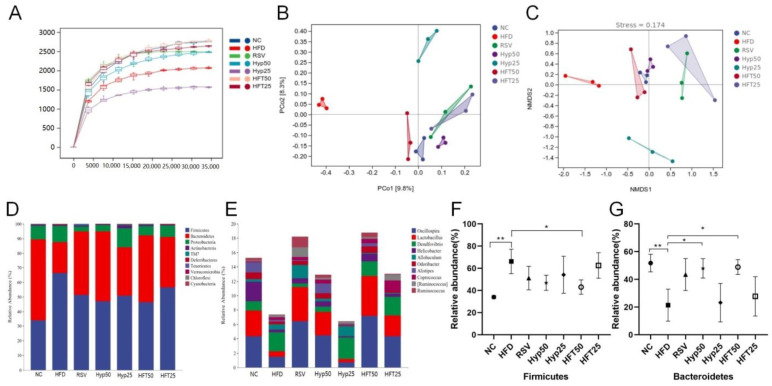
HFT affects the alpha and beta diversity and species composition of intestinal flora in ApoE^−/−^ mice. (**A**) Sparse curves, (**B**) PCoA, and (**C**) NMDS. (**D**) Phylum and (**E**) genus-level community composition. Relative abundance of (**F**) Firmicutes and (**G**) Bacteroidetes at the phylum level (*n* = 3 per group). * *p* < 0.05, ** *p* < 0.01 (correlations).

As can be seen in the sparse curve graph (Figure 3A), the curve tended to flatten more as the depth of the draw level increased. The diversities of the HFT50 and NC groups were the closest, followed by the HFT25 group, and all three were higher than that of the RSV group, while the RSV and Hyp25 groups were closer in diversity. Accordingly, HFT might regulate the abundance of flora in AS, and the effect appears to be superior to that of resveratrol calcium tablets. To evaluate beta diversity, the unweighted UniFrac distance calculation method was used to perform principal coordinate analysis (PCoA) and non-metric multidimensional scaling (NMDS). The sample points of each group were largely separated, with the HFD group differing significantly from the other groups in terms of flora structure. The NC and HFT50 groups demonstrated a high degree of intergroup similarity and relatively similar community composition (Figure 3B,C). Overall, these results indicate that AS substantially altered the structure of intestinal flora.

### 2.5. Effect of HFT on Species Composition and Variation Analysis of Intestinal Flora in ApoE^−/−^ Mice

**Figure 4:**
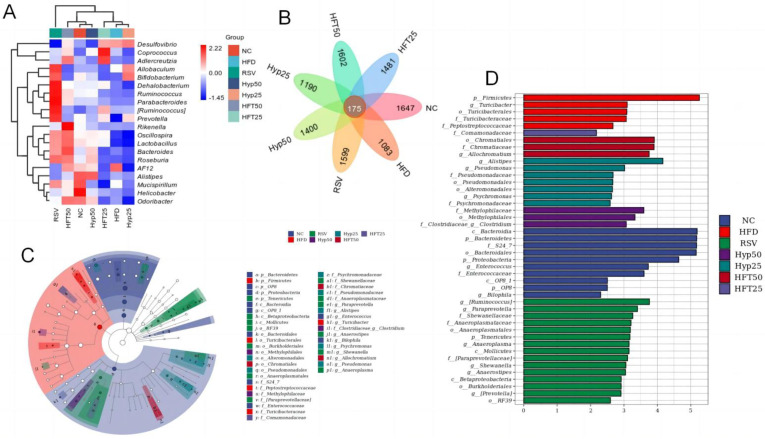
(**A**) Heatmap of the top 30 most abundant microbial species at the genus level. (**B**) Venn diagram, (**C**) LEfSe, and (**D**) cladogram. Firmicutes are represented by the red node “b”; Bacteroidetes are represented by the blue node “a”; Chromatiaceae are represented by the dark-red node “b1”; Comamonadaceae are represented by the light-purple node “y”; Clostridia are represented by the purple node “i1”; Alistipes are represented by the dark-green node “f1”; Anaerostipes are represented by the green node “j1.”.

Based on the taxonomic annotation of sequence results, the top 10 most abundant phyla were selected to assess community composition according to intragroup means of relative species abundance (Figure 3D). At the genus level (Figure 3E), the HFD group had lower relative abundances of *Oscillospira*, *Lactobacillus*, *Alistipe*, and *Odoribacter* and a higher relative *Desulfovibrio* abundance than the NC group. Following drug intervention, the relative abundances of *Oscillospira*, *Lactobacillus*, and *Allobaculum* were upregulated in the RSV, Hyp50, and HFT50 groups when compared with those of the HFD group. Compared with the NC group, the HFD group demonstrated increased abundances of Firmicutes and Proteobacteria and a decreased Bacteroidetes abundance. Compared with the HFD group, the Hyp50 and HFT50 groups exhibited reduced abundances of Firmicutes and Proteobacteria and an elevated relative abundance of Bacteroidetes (*p* < 0.05; Figure 3F,G). Based on these results, HFT might be capable of reversing AS-induced structural disorders of the intestinal flora. Species abundance distribution across samples was further assessed according to a heatmap of the top 30 most abundant species at the genus level (Figure 4A). Compared with the HFD group, *Lactobacillus* and *Bacteroides* were more abundant in the HFT50 group; *Akkermansia* and *Coprococcus* were more abundant in the HFT25 group; *Dehalobacterium* and *Ruminococcus* were more abundant in the RSV group; *Alistipes* and *Clostridium* were more abundant in the Hyp50 group; and *Bifidobacterium* and *Allobaculum* were more abundant in the Hyp25 group.

Overall, 175 species were shared among the treatment groups (Figure 4B), with 1083 exclusive to the HFD group. Following drug intervention, the number of species considerably increased across all categories, with the highest number found in the HFT50 group (1602), followed by the RSV group (1599), the HFT25 group (1481), the Hyp50 group (1400), and the Hyp25 group (1190).

Linear discriminant analysis effect size (LEfSe; Figure 4C) was assessed and, alongside a linear discriminant analysis (Figure 4D), revealed that there were ten taxa in the NC group, five taxa in the HFD group, fifteen taxa in the RSV group, three taxa each in the Hyp50 and HFT50 groups, seven taxa in the Hyp25 group, and only one taxon in the HFT25 group. At the phylum level, Firmicutes was most abundant in the HFD group, while Bacteroidetes exhibited the highest abundance in the NC group, with significant intergroup differences. At the family level, Chromatiaceae was the most abundant in the HFT50 group, and Comamonadaceae was the most abundant in the RSV group, with significant intergroup differences. At the genus level, *Clostridium* was the most abundant in the Hyp50 group. *Alistipes* and *Anaerostipes* were most abundant in the Hyp25 and RSV groups, respectively, with significant intergroup differences.

### 2.6. HFT Regulation of Lipid Metabolism in ApoE^−/−^ Mice

Based on the above findings, HFT50 could exert a marked ameliorative effect on AS. To further verify its regulatory effect on dyslipidemia and clarify the underlying mechanism of action on AS, we employed non-targeted metabolomic methods to conduct a lipidomics analysis of sera collected from the NC, HFD, and HFT50 groups. Adipose chains and groups were classified according to the data-dependent acquisition scanning mode, and the LipidSearch database was used for the analysis. Twenty-two lipid metabolites were identified, including TG, PC, sphingomyelin (SM), phosphatidylinositol (PI), lysophosphatidylcholine (LPC), cholesterol ester (CE), ceramide (Cer), diglyceride (DG), glucose ceramide (GlcCer), and lactose ceramide (LacCer). A total of 859 lipid compounds were identified.

**Figure 5:**
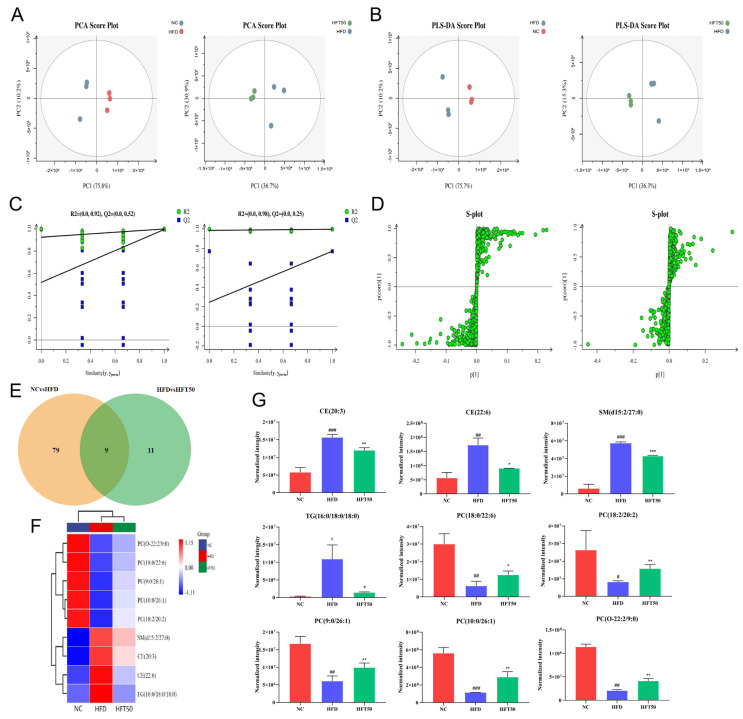
Effects of HFT on lipid metabolites in ApoE^−/−^ mice. (**A**) PCA score plots, (**B**) PLS-DA score plots, and (**C**) displacement test. (**D**) OPLS-DA permutation plot for the NC vs. HFD (left) and HFD vs. HFT50 groups (right). (**E**) Venn diagram. (**F**) Heatmap of nine differentially expressed lipids. (**G**) Histogram comparing serum differential lipid levels (*n* = 3 per group). ^###^ *p* < 0.001, ^##^ *p* < 0.01, and ^#^ *p* < 0.05 compared with the NC group; *** *p* < 0.001, ** *p* < 0.01, and * *p* < 0.05 compared with the HFD group. HFT, hybrid-mixed nanomicelles comprising Hyp in pluronic F-127 and polyethylene glycol 1000 vitamin E succinate; NC, normal control group; HFD, high-fat diet group; HFT50, HFD + high-dose HFT; PCA, principal component analysis; PLS-DA, a partial least squares-discriminant analysis; OPLS-DA, orthogonal partial least squares-discriminant analysis; PC, phosphatidylcholine; CE, cholesterol ester; SM, sphingomyelin; TG, triglyceride.

The serum metabolite data from each group of mice were categorized using principal component analysis (PCA). We found that the sample points were separated between groups, indicating that the metabolites of the two groups differed significantly from one another (Figure 5A), further suggesting that HFT could modulate AS-induced metabolic disorders. To address the potential effects of intergroup and random errors in the PCA, we performed a partial least squares-discriminant analysis (PLS-DA; Figure 5B,C) and found that the data were distributed into two relatively independent intervals between groups, indicating significant overall differences in metabolites between groups. To ensure the capacity of the model to predict and observe differences between groups in greater detail, we used markers and orthogonal PLS-DA (OPLS-DA). As shown in the S-plot (Figure 5D), the variables distributed at either end of the curve were potential biomarkers with variable importance of projection (VIP) > 1.

Based on threshold values of *p* ≤ 0.05 and VIP ≥ 1, 88 lipid levels differed significantly between the NC and HFD groups (Figure 5E). However, HFT treatment altered the levels of 20 lipids of those in the HFD group, with nine lipids significantly altered. A heatmap was plotted for the nine significantly altered lipids to better predict the biological functions of known or unknown lipids (Figure 5F). In HFD-fed mice, HFT increased and decreased the levels of five and four metabolites, respectively (Figure 5G). SM, TG, and CE were identified as key metabolites. Drug intervention reduced levels of TG (16:0/18:0/18:0), CE (20:3), CE (22:6), and SM (d15:2/27:0) (*p* < 0.05, *p* < 0.01). In a study by Raposeiras et al., an increase in TG content meant that the number of AS-impacted blood vessels also increased, resulting in vascular inflammation [37]. Drug intervention significantly reduced the TG content. Stegemann et al. have shown that CE is a detrimental factor for cardiovascular disease, and CE accumulation in foam cells could facilitate the progression of AS. Accordingly, reducing CE content could have a therapeutic effect against cardiovascular diseases, while the level could help predict cardiovascular disease [38]. These results suggested that HFT significantly ameliorated lipid metabolism disorders in mice with AS.

### 2.7. Pathway Analysis of Differentially Expressed Metabolites and Associations with Intestinal Flora

**Figure 6:**
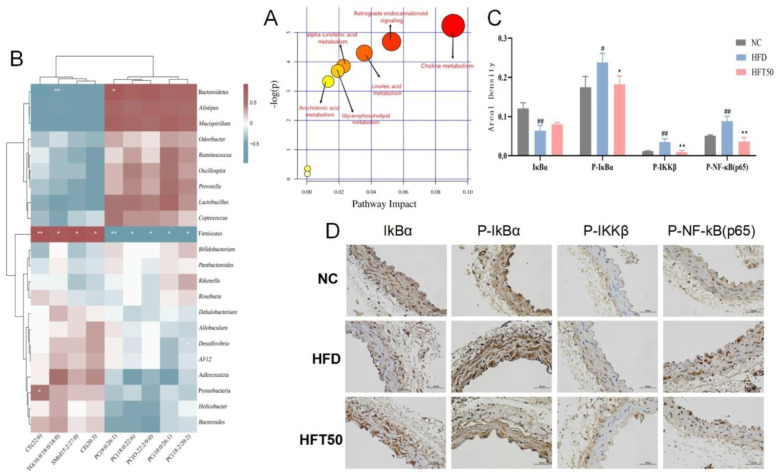
(**A**) Analysis of relevant metabolic pathways. The x-axis represents the topological analysis impact factor, and the y-axis represents the pathway enrichment analysis *p*-value (−log[P]). (**B**) Heatmap of serum lipidomics and intestinal flora. (**C**) Histogram of areal density of IκBα, P-IκBα, P-IKKβ, and P-NF-κB (p65) (*n* = 3 per group). ^##^ *p* < 0.01 and ^#^ *p* < 0.05 compared with the NC group, ** *p* < 0.01 and * *p* < 0.05 compared with the HFD group. (**D**) Histological changes in aortic tissues at a magnification of 400×.

Based on the above results, the HFD and HFT50 groups were selected for topological analysis, and all differentially expressed lipids were searched for in the Kyoto Encyclopedia of Genes and Genomes and MetaboAnalyst databases using the metabolic data platform MetaboAnalyst 5.0 (https://www.metaboanalyst.ca/MetaboAnalyst/ModuleView.xhtml (accessed on 25 June 2022)). HFT mainly regulated the cholesterol metabolism pathway in HFD-fed mice (Figure 6A).

We assessed the association between changes in the intestinal flora and lipid levels (Figure 6B) and found that Firmicutes were positively correlated with SM, TG, and CE content (*p* < 0.05, *p* < 0.01, respectively) and negatively correlated with PC content (*p* < 0.05, *p* < 0.01, respectively). Bacteroidetes were negatively correlated with TG content (*p* < 0.01) and positively correlated with PC (9:0/26:1) content (*p* < 0.05). Proteobacteria showed a positive correlation with CE (22:6) content (*p* < 0.05). Desulfovibrio was negatively correlated with PC (18:2/20:2) content (*p* < 0.05). These results are consistent with the intestinal flora analysis, demonstrating that HFT can downregulate and upregulate relative abundances of pathogenic and beneficial bacteria, respectively, further indicating that HFT attenuates AS by improving the bacterial community structure and regulating dyslipidemia.

### 2.8. HFT Regulated the NF-κB Signaling Pathway

Changes in cholesterol metabolism can lead to dyslipidemia and premature AS. AS caused by abnormal lipid function is associated with chronic inflammation [39]. The NF-κB pathway, which is closely linked to inflammation and lipid dysregulation, can be activated by SM, a vital regulator of the inflammatory response [40]. Herein, immunohistochemistry was used to detect the expression of proteins associated with the NF-κB pathway, including IκBα, P-IκBα, P-IKKβ, and P-NF-κB (p65), in vascular tissues. Compared with the NC group, the HFD group exhibited reduced IκBα expression and enhanced expression of P-IκBα, P-IKKβ, and P-NF-κB proteins (*p* < 0.05, *p* < 0.01) in aortic tissues. After HFT intervention, the expression levels of the four above-mentioned proteins tended to be similar to those of the NC group (Figure 6C,D).

## 3. Discussion

In 1904, Felix Marchand first introduced the term ‘atherosclerosis’, suggesting that AS was the cause of almost all arterial obstructive processes [41]. Disease progression can lead to the formation of AS plaques, which, in turn, narrow the arterial lumen and are characterized by lipid accumulation in the arterial wall and the infiltration of immune cells [42]. Following rupture, the plaque surface triggers thrombosis, which induces serious events such as ischemia, myocardial infarction, and cerebral infarction (ischemic stroke) [43]. Autopsy reports have described the pathology of AS as early as 500 years ago [44]. In 1908, Russian pathologists discovered that AS was caused by hypercholesterolemia in experimental animals and proposed a causal role for dietary cholesterol in AS. This experiment laid the foundation for the theory that dietary cholesterol induces AS in humans and experimental animals [45].

Hyp, a flavonol glycoside found in several Chinese herbs, has demonstrated key functions in treating cardiovascular disease, hypertension, hyperlipidemia, and inflammation, among other diseases [46,47,48]. Nanomicelles, i.e., self-assembling core-shell structures with particle sizes of 10–100 nm, afford a potential solution for enhancing the solubility of poorly soluble drugs, prolonging the duration of drug circulation, enriching drug content at lesion sites, and enhancing drug efficacy [49]. In our previous study, by combining Hyp, F127, and TPGS, we aimed to formulate HFT nanomicelles that would improve the water solubility of Hyp, prolong its circulation time in the body, and improve its bioavailability. ApoE^−/−^ mice, the animal model used in the present study, demonstrate poor lipoprotein clearance, which, in turn, leads to the accumulation of CE-rich particles in the blood, thereby promoting the development of AS [50]. Oral administration of HFT to ApoE^−/−^ mice significantly reduced the plaque area, endothelial damage, and pathological damage caused by AS in the aorta. The degree of damage reduction was greater than that observed with Hyp and rosuvastatin calcium tablets, indicating that HFT could be an effective drug for treating AS.

Furthermore, HFD can cause intestinal flora dysregulation, which may impact the progression of AS. [51,52]. We examined the mechanism through which HFT affects the modulation of inflammatory markers and the gut microbiota in relation to cholesterol metabolism. Here, we discovered that the HFD group exhibited higher abundances of Firmicutes and Proteobacteria and a lower abundance of Bacteroidetes than the NC group. Moreover, we found that an increase in Firmicute abundance and a decrease in Bacteroide abundance could trigger multiple pathways, especially the NF-κB pathway, thereby increasing the production of pro-inflammatory factors in aortic plaques [53]. HFT rescued the dysregulated Firmicute and Bacteroidete abundances, likely by reducing the levels of inflammatory factors. HFT reduced body weight and regulated blood lipid levels in HFD-fed mice, consistent with the changes in the Firmicutes/Bacteroidetes ratio. Given the strong association between body weight and blood lipid levels, these findings suggest that HFT could effectively improve the dysbiosis of the intestinal flora in mice with AS. Based on the alpha and beta diversity analyses, the total number, abundance, and distribution of gut microbiota species varied greatly between the HFD and NC groups. Cholesterol has been linked to an increase in the richness and diversity of the gut microbiota [54]. Similarly, HFT has been shown to promote the proliferation of beneficial gut microbes (e.g., *Roseburia*, *Lactobacillus*, and *Bacteroides*), thereby facilitating the production of anti-inflammatory metabolites, short-chain fatty acids, and cholesterol excretion, which were inversely associated with TG, TC, and LDL-C content. Vascular oxidative stress is critical in retaining subendothelial LDL-C, which is oxidized within inflammatory macrophages, ultimately contributing to the accumulation of foam cells and the formation of atherosclerotic plaques [55]. Accordingly, HFT significantly regulated the levels of vascular endothelial factors (iNOS and eNOS), SOD, CAT, and GSH.

It is well-established that dyslipidemia is a substantial risk factor for developing atherosclerotic cardiovascular disease [56,57]. The pathology of AS is closely related to the deposition of lipoproteins transported by blood lipids in the arterial wall [58,59]. Based on liquid chromatography-mass spectrometry (LC-MS) serum lipidomic analysis, we identified 88 differentially expressed lipids, nine of which were overlapping metabolites. These mainly included PC, SM, TG, and CE, of which SM and CE were key metabolites. These metabolites are distributed in various metabolic pathways, including cholesterol metabolism and the endocannabinoid signaling pathway. Notably, cholesterol metabolism is the main metabolic pathway for these metabolites. Cholesterol has been shown to exert an antimicrobial effect against certain intestinal microflora, inducing alterations in the microbial community [60]. Short-chain fatty acids, known substrates for cholesterol production, can be produced and metabolized by intestinal microflora to regulate various physiological functions of the host [61]. In the HFD group, the serum levels of SM and CE were significantly increased when compared with those in the NC group, and the SM and CE levels were positively correlated with Firmicute and Proteobacteria abundance, respectively (*p* < 0.05, *p* < 0.01). HFT effectively reversed the increase in SM and CE levels and enhanced the abundance of beneficial bacteria.

Multiple studies have confirmed the important role of beneficial bacteria in regulating the NF-κB pathway [62,63,64]. As a key factor affecting obesity-induced insulin resistance and intestinal flora-mediated inflammation, activating the SM metabolic pathway can promote the phosphorylation of IKK, which activates the NF-κB pathway by phosphorylating IκBα [65]. Inflammation-related proteins are expressed more frequently as a result of NF-κB release and IκBα phosphorylation, and NF-κB activation contributes to the production of pro-inflammatory factors, thereby resulting in AS. All of this is consistent with the experimental results [5,66,67,68]. The expression levels of P-IκBα, P-IKKβ, and P-NF-κB (p65) were significantly upregulated in the aorta of HFD mice, whereas IκBα expression was significantly downregulated. HFT intervention significantly reduced the phosphorylation of the IκBα protein and downregulated P-NF-κB (p65) expression in the nucleus. These findings suggest that HFT restricted the movement of the NF-κB p65 subunit from the cytoplasm to the nucleus, thereby inhibiting the inflammatory process. Therefore, the beneficial effect of HFT on AS may be related to the elevated production of beneficial metabolites and alterations in the microbiome, thereby enhancing the abundance of beneficial bacteria, which produce various short-chain fatty acids that affect the activation of the NF-κB signaling pathway.

## 4. Materials and Methods

### 4.1. Materials and Reagents

Hyp (AF2020041503) was purchased from Chengdu Alfa Biotechnology (Chengdu, China); F127 (RA0213008) and TPGS (RJ0210731) were purchased from Xi’an Ruixi Biotechnology (Xi’an, China); enzyme-linked immunosorbent assay (ELISA) kits for IL-7, IL-6, IL-1β, GSH, CAT, TNF-β, TNF-α, eNOS, iNOS, and SOD were obtained from Jiangsu Kete Biotechnology (Yancheng, China).

### 4.2. Preparation of HFT

HFT was prepared using a thin-film dispersion method by dissolving 9 mg Hyp as well as F127 and TPGS (2:1) in acetone via ultrasonication (Kunshan Ultrasonic Instrument, Shanghai, China) for 5 min and performing a rotary evaporation (Shanghai Yarong Biochemistry Instrument Factory, Shanghai, China) under reduced pressure at 45 °C until a uniform honeycomb-like film formed on the flask wall. The flask was then placed in a vacuum desiccator (at room temperature) and incubated overnight. Next, 3 mL of phosphate-buffered saline (PBS, pH = 7.4 ± 0.1) was added, which was followed by the hydration of the solution for 2 h at 50 °C with continuous stirring. The solution was centrifuged at 4 °C (10,000 r min^−1^, 10 min), and the supernatant was extracted.

### 4.3. Experimental Animals

Forty-eight ApoE^−/−^ and 8 C57BL/6 male mice (8 weeks old, 20–25 g) were purchased from Changzhou Cavins Laboratory Animal (License No.: SCXK [Su] 2016-0010). All mice were maintained at 22 ± 2 °C in a specified pathogen-free (SPF)-level laboratory (relative humidity: 50 ± 10%) with a cycle of light and dark lasting 12 h and given unlimited access to food and drink at the animal center of the Changchun University of Chinese Medicine (CCUCM). Seven groups of mice were formed at random after one week of adaptive feeding with normal feed (NC group), an HFD (HFD group; Jiangsu Medicience Biotechnology, Yangzhou, China), HFD + resveratrol (RSV group; Jiangsu Simcere Pharmaceutical, Nanjing, China), HFD + high-dose Hyp (Hyp50 group), low-dose Hyp (Hyp25 group), HFD + high-dose HFT (HFT50 group), or low-dose HFT (HFT25 group). The RSV treatment was administered at a dose of 10 mg kg^−1^, and the Hyp and HFT treatments were administered at 50 and 25 mg kg^−1^ by gavage once daily, while equal volumes of saline were administered to the NC and HFD groups. The body masses of the mice were measured weekly from the start of acclimatization to the end of the trial.

### 4.4. Sample Collection

Before the experiment, the mice were fasted for 12 h without water, weighed, and anesthetized with 2% sodium pentobarbital, and their apical blood was sampled. The collected blood was placed in 1.5 mL EP tubes, rested (1–2 h), and centrifuged (4 °C, 3500 r min^−1^, 15 min), and the supernatant was removed and stored (–80 °C) as a backup. The harvested aorta was placed in a pre-cooled 6 cm Petri dish with PBS solution. After separating the surrounding connective tissues under a microscope (Olympus, Tokyo, Japan), the aortic tissue was fixed in a 10 mL EP tube containing 4% paraformaldehyde (Beijing Coolaber Technology, Beijing, China) for subsequent embedding. Meanwhile, the contents of the mouse cecum were placed in dry, sterilized, lyophilized tubes at –80 °C for subsequent sequencing analysis of the intestinal flora.

### 4.5. Aortic Plaque Analysis

To prepare aortic samples, paraffin sections (5 μm) were cut, dewaxed, stained with H and E, dehydrated, cleared for imaging, and sealed. Data analysis and processing were performed, and relative plaque area (%) = plaque area/lumen area × 100.

### 4.6. Detection of Blood Lipid Levels

The serum samples were centrifuged (Sigma-Aldrich, Darmstadt, Germany) again for 15 min at 4 °C and 3500 r min^−1^ to collect the supernatant while avoiding hemolysis. Then, 200 μL of each sample was used to detect the serum levels of TG (20190925), TC (20190918), LDL-C (20190923), and HDL-C (20190922) using a fully automated biochemical analyzer (Selectra Junior, Rittal, Zevenaar, The Netherlands). For assessment, three serum samples were randomly selected from each group of mice (using the corresponding ELISA kits) to establish the levels of IL-6, IL-7, IL-1β, iNOS, CAT, TNF-α, TNF-β, eNOS, GSH, and SOD.

### 4.7. 16S rRNA Amplification and Sequencing to Detect Intestinal Flora

We randomly selected 21 cecal content samples (three from each group) and extracted the total genomic DNA according to the instructions of the DNA extraction kit (Guangzhou Magen Biotechnology, Guangzhou, China). Fluorescence spectrophotometer measurements of DNA concentration were performed at 260 and 280 nm (E6090, Promega, Madison, WI, USA), along with 1% agarose gel electrophoresis (DYY-6C, Beijing Liuyi Biotechnology, Beijing, China) for assessing DNA quality. The primers 338F (5′-ACTCCTACGGGAGGCAGCA-3′) and 806R (5′-GGACTACHVGGGTWTCTAAT-3′) were used to amplify the V3–V4 region of the 16S rRNA gene using a PCR system (Bio-Rad Laboratories, Hercules, CA, USA). At last, sequence libraries were constructed using a standard Illumina TruSeq DNA library preparation procedure. The Illumina platform (Illumina, San Diego, CA, USA) was utilized for sequencing, and the DADA2 approach was used for depriming, mass filtering, denoising, splicing, and chimera removal. Each deduplicated sequence generated after quality control was termed either an amplicon sequence variant or signature sequence (corresponding to the operational taxonomic unit-representative sequences), and data analysis was performed using Genescloud tools (https://www.genescloud.cn/home (accessed on 18 June 2022)).

### 4.8. Serum Lipidomic Analysis

Briefly, serum was added to a chloroform–methanol mixture and vortexed for 30 s. The serum was placed on ice, vortexed with ultra-pure water for 30 s, and left to rest. The lower layer was centrifuged at room temperature, transferred to a new 2 mL EP tube, added to the chloroform–methanol mixture, and vortexed for 30 s. The lower layer was concentrated using a vacuum centrifuge (5305, Eppendorf, Hamburg, Germany). Isopropyl alcohol was added to obtain a sample for LC-MS detection. The chromatographic conditions were identical to those used in a previous study.

### 4.9. Immunohistochemical Analysis

Aortic sections were submerged in 3% bovine serum albumin, treated with a primary antibody (incubated overnight at 4 °C), treated with a secondary antibody, and incubated for 50 min. Nuclei were then re-stained with hematoxylin, blocked, and examined under a microscope (E100, Nikon, Tokyo, Japan). Images were acquired and analyzed using an ortho-white photomicroscope (Nikon, Japan). We measured the cumulative optical density (IOD) values of three positive fields of view in each section separately and calculated the corresponding tissue pixel areas (area) and areal density (IOD/area). All reagents were provided by Wuhan Servicebio Technology (Wuhan, China).

### 4.10. Statistical Analysis

All statistical analyses and graphics generation were performed using SPSS (version 20.0; IBM SPSS, Armonk, NY, USA) and GraphPad Prism 8 software (GraphPad Software, San Diego, CA, USA). Data are expressed as the mean ± standard deviation (±SD). For normally distributed data, a one-way analysis of variance was used for multiple group comparisons, and the c was used for comparisons between groups. Differences were considered statistically significant at *p* < 0.05.

## 5. Conclusions

Collectively, our findings revealed that HFT could rescue the aorta from pathological damage, regulate lipid levels, inhibit inflammatory factor expression, reduce oxidative damage, and improve gut flora community structure, ultimately attenuating the AS-related effects present in AopE^−/−^ mice. HFT reversed the expression of various abnormally altered flora in AopE^−/−^ mice to achieve similar levels as those observed in the NC group. Our study demonstrates the potential mechanism through which HFT could regulate dyslipidemia and inflammatory damage during AS treatment, validating the results of the intestinal flora study. Simultaneously, we hypothesized that HFT could improve the disordered gut microbiota structure by regulating cholesterol metabolism pathways, elevating PC levels, and reducing those of TG, SM, and CE. Moreover, the mechanism through which HFT alleviates AS might involve modulating NF-κB-related protein expression and reducing inflammatory damage. This research offers an empirical foundation for the therapeutic potential of Hyp and the development and application of new dosage forms. Moreover, our findings could generate novel ideas regarding the anti-inflammatory mechanisms of other drugs. Although this study provides a preliminary analysis of the underlying mechanism of action from the perspective of intestinal flora and serum lipidomics, unresolved challenges persist, and in-depth research and development endeavors are needed to ensure promising applications in additional fields.

## Figures and Tables

**Table 1:** Effect of HFT on the alpha diversity of intestinal flora.

Group	Chao1	Observed Species	Shannon	Simpson
NC	2757.43 ± 181.31	2177.30 ± 94.20	7.94 ± 0.30	0.97 ± 0.01
HFD	2068.69 ± 304.76 ^a^	1706.27 ± 295.53	7.26 ± 1.07	0.94 ± 0.06
RSV (10 mg/kg)	2480.32 ± 400.95	2253.87 ± 422.73	8.65 ± 0.47	0.99 ± 0.01
Hyp50 (50 mg/kg)	2478.79 ± 151.82	2015.27 ± 109.25	7.80 ± 0.28	0.96 ± 0.01
Hyp25 (25 mg/kg)	1564.13 ± 351.88	1350.40 ± 330.07	7.62 ± 1.07	0.92 ± 0.08
HFT50 (50 mg/kg)	2781.23 ± 445.59	2241.73 ± 440.72	8.32 ± 0.25	0.97 ± 0.03
HFT25 (25 mg/kg)	2633.15 ± 363.02	2245.87 ± 259.96	8.10 ± 0.91	0.93 ± 0.05

Values are presented as the mean ± standard deviation (SD) (*n* = 3 per group). ^a^ *p* < 0.05 vs. NC group.

## Data Availability

Data is contained within the article or Supplementary Material.

## Supplementary Materials

The following supporting information can be downloaded at: https://www.mdpi.com/article/10.3390/molecules28135088/s1. Table S1. Effects of HFT on aortic intimal plaque area in ApoE^−/−^ mice. Table S2. Effects of HFT on blood lipid levels in ApoE^−/−^ mice. Table S3. The effects of ASBUE on pro-inflammatory markers in ApoE^−/−^ mice. Table S4. Effects of ethyl acetate and n-butanol extract of Acanthopanax senticosus on serum eNOS, iNOS, SOD, CAT and GSH levels in ApoE^−/−^ mice. Table S5. Effects of HFT on intestinal microflora F/B in ApoE^−/−^ mice. Table S6. List of lipid difference between NC group and HFD group. Table S7. List of lipid difference between HFD group and HFT50 group.

## Sample Availability

Samples are available from the authors upon request.
