# Supplementary material for: Hyperoside Nanomicelles Alleviate Atherosclerosis by Modulating the Lipid Profile and Intestinal Flora Structure in High-Fat-Diet-Fed Apolipoprotein-E-Deficient Mice
Source: Molecules. 2023 Jun 29;28(13):5088. doi: 10.3390/molecules28135088 (PMC10343736; doi:10.3390/molecules28135088)
Supplement: Supplementary file 1 [file molecules-28-05088-s001.zip › molecules-2420843-supplementary.pdf]

Table S1. Effects of HFT on aortic intimal plaque area in ApoE$^{-/-}$ mice

Group	Plaque Areas
NC	0.00 ± 0.00
HFD	31.27 ± 4.72^{b}
RSV (10 mg/kg)	17.74 ± 1.72^{c}
Hyp (50 mg/kg)	8.33 ± 0.77^{d}
Hyp (25 mg/kg)	9.83 ± 3.85^{c}
HFT (50 mg/kg)	5.07 ± 0.82^{d}
HFT (25 mg/kg)	7.22 ± 1.42^{d}

Values are presented as the mean ± SD. N = 3 in each group. $^{a}P<0.05$，$^{b}P<0.01$ vs. NC group; $^{c}P<0.05$，$^{d}P<0.01$ vs. HFD group.

Table S2. Effects of HFT on blood lipid levels in ApoE$^{-/-}$ mice

Group	TG/mmol·L^{-1}	TC/mmol·L^{-1}	LDL-C/mmol·L^{-1}	HDL-C/mmol·L^{-1}
NC	0.80 ± 0.29	3.46 ± 1.61	2.06 ± 0.79	1.60 ± 0.07
HFD	1.90 ± 0.21^{b}	32.60 ± 4.18^{b}	11.00 ± 2.30^{b}	0.29 ± 0.04^{b}
RSV (10 mg/kg)	1.06 ± 0.42^{c}	23.98 ± 0.47^{c}	8.64 ± 2.06	1.09 ± 0.19^{d}
Hyp (50 mg/kg)	1.09 ± 0.36^{c}	28.61 ± 1.17	7.96 ± 0.88	1.19 ± 0.05^{d}
Hyp (25 mg/kg)	1.64 ± 0.84	30.67 ± 7.89	10.41 ± 1.83	0.91 ± 0.26^{c}
HFT (50 mg/kg)	0.84 ± 0.24^{d}	21.70 ± 1.33^{c}	5.22 ± 0.10^{c}	1.53 ± 0.44^{d}
HFT (25 mg/kg)	0.96 ± 0.49^{c}	27.32 ± 5.72	8.60 ± 2.27	1.12 ± 0.16^{d}

Values are presented as the mean ± SD. N = 3 in each group. $^{a}P<0.05$，$^{b}P<0.01$ vs. NC group; $^{c}P<0.05$，$^{d}P<0.01$ vs. HFD group.

TableS3. The effects of ASBUE on pro-inflammatory markers in ApoE-/- mice

Group	IL-1β/ng·L^{-1}	IL-6/pg·mL^{-1}	IL-7/ng·L^{-1}	TNF-α/ng·L^{-1}	TNF-β/ng·L^{-1}
NC	34.21±5.46	94.61±1.68	12.05±1.67	407.95±98.81	141.84±17.19
HFD	61.30±8.40^{b}	122.94±11.52^{b}	18.76±3.25^{b}	794.23±59.63^{b}	185.20±11.66^{a}
RSV (10 mg/kg)	41.22±9.12^{c}	108.42±8.72	12.62±2.84^{c}	628.85±65.71^{d}	154.26±11.97^{c}
Hyp	46.41±3.97^{c}	109.61±3.96	10.86±1.38^{d}	728.59±49.41	147.96±7.95^{d}

(50 mg/kg)					
Hyp (25 mg/kg)	51.15±7.81	112.06±6.08	14.39±2.87	789.94±95.68	156.07±3.36^{d}
HFT (50 mg/kg)	38.08±3.20^{c}	96.72±5.75^{c}	10.29±0.33^{d}	611.92±40.69^{d}	133.26±9.23^{d}
HFT (25 mg/kg)	40.77±5.92^{c}	100.66±5.29^{c}	11.67±3.22^{c}	705.05±75.50	149.02±14.34^{c}

Values are presented as the mean ± SD. N = 3 in each group. ^{a}P<0.05，^{b}P<0.01 vs. NC group; ^{c}P<0.05，^{d}P<0.01 vs. HFD group.

Table S4. Effects of ethyl acetate and n-butanol extract of Acanthopanax senticosus on serum eNOS, iNOS, SOD, CAT and GSH levels in ApoE-/- mice

Group	iNOS/μmol·L^{-1}	eNOS/μmol·L^{-1}	SOD/pg·mL^{-1}	CAT/ng·L^{-1}	GSH/ng·L^{-1}
NC	10.30±2.19	9.02±0.74	68.31±11.01	42.61±3.62	151.71±12.90
HFD	16.02±2.91^{a}	5.93±0.63^{b}	36.10±7.41^{b}	29.25±3.00^{b}	87.81±3.95^{b}
RSV (10 mg/kg)	12.17±1.79^{c}	8.31±0.27^{d}	54.01±9.03^{d}	37.13±3.53^{c}	115.38±24.51
Hyp (50 mg/kg)	11.04±0.64^{c}	8.30±0.07^{d}	55.59±6.48^{d}	38.72±2.25^{c}	107.10±22.75
Hyp (25 mg/kg)	12.46±1.08^{c}	7.15±1.40	43.18±10.72	31.98±5.53	92.58±29.51
HFT (50 mg/kg)	9.79±1.91^{c}	9.15±0.63^{d}	63.77±17.00^{d}	43.67±4.84^{c}	149.04±5.34^{d}
HFT (25 mg/kg)	10.65±1.65^{c}	7.63±0.23^{d}	58.73±11.52^{d}	38.44±1.54^{d}	103.75±13.09

Values are presented as the mean ± SD. N = 3 in each group. ^{a}P<0.05，^{b}P<0.01 vs. NC group; ^{c}P<0.05，^{d}P<0.01 vs. HFD group.

Table S5. Effects of HFT on intestinal microflora F/B in ApoE$^{-/-}$ mice

Group	Firmicutes/%	Bacteroidetes/%	F/B

NC	34.01±1.38	51.74±6.34	0.66±0.09
HFD	66.18±11.00^{b}	21.38±11.58^{b}	3.79±1.96^{b}
RSV (10 mg/kg)	51.24±10.58	43.52±11.46	1.30±0.69
Hyp (50 mg/kg)	46.98±6.82	47.97±6.98^{c}	1.01±0.28
Hyp (25 mg/kg)	50.82±22.39	33.21±30.69	2.96±2.34
HFT (50 mg/kg)	43.05±6.48^{c}	48.89±5.40^{c}	0.90±0.22
HFT (25 mg/kg)	62.54±11.51	27.75±14.18	2.91±2.05

Values are presented as the mean ± SD. N = 3 in each group. ^{a}P<0.05，^{b}P<0.01 vs. NC group; ^{c}P<0.05，^{d}P<0.01 vs. HFD group.

Table S6. List of lipid difference between NC group and HFD group

Name	Class	m/z	RT	FC	*P*-value
PC(10:0/26:1)	PC	788.6108	547.51	4.8827	0.0003
PC(16:0/18:1)	PC	804.5797	480.62	2.0123	0.0003
PC(18:2/22:6)	PC	830.5607	359.03	0.3136	0.0004
PC(2:0/30:0)	PC	734.5633	476.35	4.9623	0.0007
PC(24:4/14:0)	PC	810.5921	555.01	3.2641	0.0011
PC(9:0/26:1)	PC	774.5910	515.86	2.7511	0.0018
PC(2:0/32:0)	PC	762.5955	547.90	5.6113	0.0019
PC(4:0/30:0)	PC	762.5858	480.44	2.5165	0.0030
PC(18:0/22:6)	PC	856.5717	460.89	0.2057	0.0032
PC(14:0/22:5)	PC	780.5490	377.88	2.7572	0.0037
PC(14:1/24:4)	PC	808.5778	418.89	0.5806	0.0050
PC(7:0/26:1)	PC	746.5610	453.90	2.8340	0.0062
PC(22:5/16:1)	PC	828.5418	399.86	0.2504	0.0090
PC(22:2/18:4)	PC	878.5936	459.97	0.4194	0.0094

PC(16:0/18:2)	PC	802.5638	429.56	0.5645	0.0095
PC(14:0/24:4)	PC	810.5945	471.85	0.6515	0.0095
PC(16:1/24:4)	PC	836.6034	476.87	0.4526	0.0118
PC(18:0/20:4)	PC	854.5952	471.23	0.5863	0.0119
PC(22:6/16:0)	PC	850.5633	398.57	0.6216	0.0249
PC(18:0/18:1)	PC	832.6102	547.82	3.2293	0.0250
PC(6:0/26:1)	PC	732.5472	421.57	4.0629	0.0391
PC(18:2/20:2)	PC	810.5817	419.49	0.3115	0.0471
TG(17:0/17:0/17:1)	TG	864.8194	741.31	70.6070	0.0000
TG(15:1/20:5/18:1)	TG	880.7336	686.48	0.0796	0.0000
TG(15:1/20:5/18:2)	TG	878.7171	668.82	0.1041	0.0001
TG(18:2/16:0/18:2)	TG	872.7609	668.62	0.1044	0.0006
TG(16:0/16:1/18:2)	TG	846.7431	665.91	0.1771	0.0040
TG(18:2/15:1/20:4)	TG	880.7277	692.50	0.1550	0.0040
TG(16:0/18:2/18:2)	TG	872.7558	675.11	0.1800	0.0042
TG(16:1/18:2/18:2)	TG	870.7452	654.98	0.2050	0.0043
TG(16:1/16:0/18:1)	TG	848.7608	684.07	0.2068	0.0048
TG(14:0/18:2/18:2)	TG	844.7287	651.10	0.2096	0.0055
TG(18:1/16:0/18:1)	TG	876.7921	703.00	0.3341	0.0091
TG(16:0/18:0/18:0)	TG	880.8188	750.95	37.8860	0.0102
TG(16:0/22:6/18:2)	TG	920.7623	652.62	0.2710	0.0116
TG(18:1/18:3/18:3)	TG	894.7452	646.18	0.2479	0.0124
TG(16:0/18:1/18:2)	TG	874.7757	688.26	0.2383	0.0157
TG(18:1/18:1/18:2)	TG	900.7902	687.60	0.2113	0.0177
TG(18:1/18:2/18:1)	TG	900.7820	669.50	0.3134	0.0259
TG(20:4/18:0/20:4)	TG	948.7873	672.53	0.3457	0.0264
TG(16:0/16:0/18:2)	TG	848.7552	689.87	0.3737	0.0281
TG(18:2/18:2/20:3)	TG	922.7673	648.69	0.3705	0.0307
TG(18:1/18:1/20:3)	TG	926.8018	694.44	0.4105	0.0367
TG(18:1/22:6/18:2)	TG	946.7746	655.66	0.3775	0.0433

SM(d15:2/27:0)	SM	857.6778	613.31	7.1175	0.0002
SM(d14:1/22:1)	SM	729.5820	412.63	5.6483	0.0013
SM(d14:0/26:0)	SM	789.6664	604.17	8.1833	0.0016
SM(d14:2/26:1)	SM	783.6254	482.01	0.1700	0.0044
SM(d19:1/27:0)	SM	871.7485	655.28	0.2121	0.0045
SM(d21:1/27:0)	SM	899.7777	674.27	0.1945	0.0050
SM(d14:0/20:0)	SM	705.5806	437.65	9.2482	0.0072
SM(d14:0/26:1)	SM	831.6629	605.57	3.7236	0.0093
SM(d16:0/26:1)	SM	815.6897	675.98	7.3510	0.0121
SM(d15:0/27:0)	SM	817.6962	676.52	7.1508	0.0128
SM(d21:0/27:0)	SM	901.7936	687.53	0.2164	0.0158
SM(d16:1/26:1)	SM	857.6779	592.09	6.2598	0.0226
SM(d21:2/27:0)	SM	897.7633	662.27	0.3245	0.0281
SM(d15:1/27:0)	SM	859.6930	688.31	10.9400	0.0473
SM(d14:1/26:0)	SM	787.6632	605.29	5.8500	0.0489
PC(P-22:0/15:0)	PC(P)	788.6659	605.27	5.3905	0.0001
PC(P-22:0/22:5)	PC(P)	876.7001	654.78	0.1922	0.0004
PC(P-22:0/17:1)	PC(P)	814.6823	599.77	10.2200	0.0013
PC(P-22:0/16:0)	PC(P)	802.6784	651.52	4.9158	0.0014
PC(P-22:0/14:1)	PC(P)	772.6155	559.17	17.2930	0.0058
PC(P-22:0/17:0)	PC(P)	816.6835	599.78	8.9676	0.0067
PC(P-20:0/18:3)	PC(P)	796.6136	531.45	16.4710	0.0172
PC(P-22:0/9:0)	PC(P)	704.5731	400.47	11.9760	0.0448
CE(20:3)	CE	692.6250	693.60	0.3697	0.0004
CE(20:4)	CE	690.6124	672.84	0.2657	0.0012
CE(18:1)	CE	668.6292	707.51	9.0782	0.0031
CE(22:6)	CE	714.6127	661.36	0.3273	0.0031
CE(16:1)	CE	640.5952	679.96	4.8930	0.0102
CE(18:3)	CE	664.5981	663.49	15.8700	0.0166
CE(18:0)	CE	670.6409	736.74	67.4460	0.0242

CE(20:5)	CE	688.5974	652.76	0.4857	0.0284
PC(O-22:2/9:0)	PC(O)	702.5586	359.05	2.0073	0.0029
PC(O-20:2/20:4)	PC(O)	820.6106	509.48	4.7609	0.0074
PC(O-16:2/22:4)	PC(O)	792.5808	446.80	4.4606	0.0114
PC(O-22:2/12:0)	PC(O)	744.5815	485.76	4.1734	0.0295
LPC(18:2/0:0)	LPC	542.3163	90.58	0.3873	0.0094
LPC(16:0/0:0)	LPC	518.3167	95.85	0.3883	0.0102
LPC(22:6/0:0)	LPC	590.3176	72.49	0.4713	0.0167
Sph(d17:0)	Sph	288.2900	74.20	0.4507	0.0096
Sph(d19:0)	Sph	316.3194	100.14	0.4281	0.0100
GlcCer(d18:1/16:0)	GlcCer	700.5672	361.68	29.5880	0.0000
GlcCer(d18:2/18:2)	GlcCer	722.5492	361.72	12.9090	0.0201
PI(18:0/20:3)	PI	887.5682	353.77	4.7896	0.0002
LPE(20:4/0:0)	LPE	500.2810	73.51	0.3340	0.0081

Table S7. List of lipid difference between HFD group and HFT50 group

Name	Class	m/z	RT	FC	*P*-value
SM(d15:2/27:0)	SM	857.6778	613.31	1.3251	0.0001
PC(O-22:2/9:0)	PC(O)	702.5586	359.05	2.7485	0.0001
CE(18:2)	CE	666.6140	685.85	1.8173	0.0004
CE(20:3)	CE	692.6250	693.60	2.0702	0.0022
PI(18:1/20:3)	PI	904.5836	325.84	1.5058	0.0032
PC(18:2/20:2)	PC	810.5817	419.49	1.9281	0.0049
PC(10:0/26:1)	PC	788.6108	547.51	0.5153	0.0066
PC(9:0/26:1)	PC	774.5910	515.86	0.5927	0.0082
TG(16:0/18:0/18:0)	TG	880.8188	750.95	0.1285	0.0150
SM(d20:2/22:1)	SM	855.6619	543.25	1.5141	0.0197
SM(d14:0/22:0)	SM	733.6039	469.75	1.5646	0.0200
PI(18:0/18:1)	PI	863.5679	366.85	0.4593	0.0239
Cer(t18:0/24:0)	PhytoCer	668.6483	558.94	0.4985	0.0264

TG(18:3/18:2/19:0)	TG	912.7972	758.59	0.1345	0.0338
PC(18:0/22:6)	PC	856.5717	460.89	2.0236	0.0375
CE(22:6)	CE	714.6127	661.36	1.5990	0.0388
PC(28:0/4:0)	PC	778.5648	475.72	1.2578	0.0447
TG(18:1/18:1/20:0)	TG	932.8589	752.09	0.2701	0.0467
SM(d14:1/22:0)	SM	753.5786	467.29	1.8291	0.0483
PC(14:0/20:3)	PC	756.5475	390.56	0.5055	0.0487
